# The Deep Darkness microbiome: functional and taxonomic diversity in an oligotrophic temperate cave

**DOI:** 10.1128/spectrum.01509-26

**Published:** 2026-06-30

**Authors:** Philips O. Akinwole, Nina G. Shaffer, Emma E. C. Jacobs, Zoe Green, Thi Doan, Crimson Hickman, Kaija M. Carr, Kenneth L. Brown

**Affiliations:** 1 Biology Department, DePauw University 3608, Greencastle, Indiana, USA; 2 Ecology and Evolutionary Biology Department, Tulane University 5783, New Orleans, Louisiana, USA; 3 Leighton School of Nursing, Marian University 4053 , Indianapolis, Indiana, USA; 4 Department of Geology and Environmental Geosciences, DePauw University 3608, Greencastle, Indiana, USA; Connecticut Agricultural Experiment Station, New Haven, Connecticut, USA

**Keywords:** microbial communities, oligotrophic, Deep Darkness, limestone cave, community-level physiological profiles, microbial diversity

## Abstract

Many subterranean ecosystems are chronically energy limited, yet the mechanisms governing microbial community assembly and metabolic function under extreme carbon scarcity remain poorly resolved. We combined 16S rRNA gene sequencing with community-level physiological profiling (CLPP) to examine how physicochemical gradients regulate microbial diversity and carbon-use strategies across a surface-subsurface transition into the Deep Darkness zone of Indiana Caverns. Progressive isolation from surface inputs resulted in sharp declines in microbial biomass, total organic carbon, and nitrogen, accompanied by increased water content and C:N ratios, indicating strong attenuation of allochthonous organic matter and intensified resource filtering. Microbial communities spanned 42 bacterial phyla, but exhibited compositional shifts consistent with selection for carbon-efficient and non-heterotrophic metabolisms. Cave sediments were dominated by Proteobacteria (47.6%), Acidobacteria (13.6%), Chloroflexi (9.1%), and Nitrospirae (8.3%). In contrast, Actinobacteria, typically abundant in oligotrophic caves, were exceptionally rare (1.2%), suggesting that extreme depletion of refractory organic substrates constrains decomposer-based energy acquisition and favors taxa adapted to chemolithotrophy or resource-conserving strategies. Despite severe carbon limitation, cave communities retained high phylogenetic richness, but were strongly structured by sediment chemistry and moisture availability, indicating niche differentiation driven by environmental filtering rather than passive dispersal. Only 19.8% of operational taxonomic units (OTUs) were shared across all sites, consistent with a small, persistent core microbiome. CLPP analyses further revealed functional reorganization: surface reference soil preferentially oxidized labile substrates, whereas cave communities relied more heavily on recalcitrant and polymeric carbon sources. Together, these results demonstrate that extreme oligotrophy restructures cave microbiomes around resource-efficient metabolic guilds shaped by hydrological and geochemical constraints.

**IMPORTANCE:**

Caves provide natural laboratories for understanding how microbial communities persist under extreme energy limitation. Yet, the mechanisms linking subterranean physicochemistry with microbial functional capacity remain largely unresolved. By integrating culture-independent sequencing with metabolic profiling across spatial and hydrological gradients, this study shows how carbon scarcity, sediment stoichiometry, and microhabitat structure filter microbial taxa and select for specialized metabolic guilds. The work highlights that subterranean environments can harbor high microbial diversity despite chronic oligotrophy, and that functional potential shifts predictably toward degradation of complex substrates under nutrient scarcity. The unusually low abundance of Actinobacteria, typically dominant in oligotrophic caves, highlights a distinct subterranean energy regime that favors slow-growing, resource-efficient taxa. Our findings provide new insight into how environmental filtering, hydrologic connectivity, and metabolic specialization structure microbiomes in deep karst systems, informing broader models of microbial survival in low-energy environments.

## INTRODUCTION

Subterranean environments like caves constitute compelling examples of extreme ecosystems; environments that defy traditional paradigms of microbial ecology by combining perpetual darkness, low nutrient availability, stable temperatures, and high humidity (1, 2). Far removed from the reach of sunlight, these subterranean ecosystems rely entirely on limited organic inputs, such as seepage of dissolved organic carbon, occasional particulate deposition, bat guano, or mineral-associated carbon (3–6). In the absence of photosynthesis, cave microbial life is sustained by resource economies that favor metabolic specialization and energy efficiency; a stark contrast to surface microbial communities, which often thrive on abundant labile carbon (2). In these nutrient-poor environments, microbial communities face perpetual energy limitation. This ecological niche fosters unique adaptations: slower growth rates, flexible metabolism, and the capacity to exploit complex or recalcitrant carbon sources (3). However, microbial persistence in cave systems offers invaluable insight into life’s resilience under chronic resource scarcity and analogous conditions found in deep subsurface habitats (2, 7) or other remote ecosystems on Earth and potentially beyond.

Investigations across diverse cave systems worldwide have revealed remarkable microbial diversity and metabolic potential, even in environments of extreme oligotrophy. For instance, subarctic caves in boreal latitudes, characterized by near-freezing temperatures and organic paucity, were found to harbor more diverse and complex bacterial communities compared to adjacent reference soil, highlighting resource scarcity as a driver of niche partitioning and community diversification (7). In tropical karst environments, where external nutrient inputs may vary seasonally or with rainfall patterns, microbial biomass and community dynamics closely tracked the availability of organic matter, underscoring the sensitivity of cave microbiota to resource pulses (5, 8). Studies of cave ecosystems with minimal human disturbance in temperate Europe have similarly demonstrated that microbial substrate preferences and community composition are tightly linked to the availability and chemical complexity of organic inputs (6). In North America, research in temperate-zone karst systems, such as in the Appalachians and Ozarks, has elucidated how microbial assemblages in caves display distinctive taxonomic and functional signatures compared to overlying soils, and how these assemblages contribute to subterranean nutrient cycling and mineral weathering (9–11). Collectively, these studies suggest a global trend: despite low biomass and energy input, cave microbial communities are metabolically versatile, often dominated by taxa capable of degrading complex substrates. Their functional attributes appear to be shaped by long-term adaptation to nutrient scarcity, hydrological connectivity, and habitat stability (1, 3). The distribution and functional potential of microbial communities in cave ecosystems are strongly shaped by hydrological connectivity, which governs the delivery of organic matter and nutrients from the surface (12, 13). In the Binkley Cave system in this study, surface-to-subsurface connectivity is inferred from the established karst hydrogeology of the Mitchell Plateau, where the underground river of the Deep Darkness section is known to discharge at Harrison Spring on Blue River (14), and is further supported by the moderate conductivity values measured at our sampling sites (458–572 µS/cm), consistent with autogenic epikarst recharge rather than isolated stagnant water (15). No direct hydrological tracer tests were conducted in this study; connectivity is therefore treated as an inference from published hydrogeological surveys and *in situ* water chemistry rather than as an independently verified finding. Where hydrological inputs are active, episodic influxes of dissolved organic carbon and fine sediments can stimulate microbial metabolism and broaden functional diversity, whereas more isolated zones remain subject to extreme oligotrophy (16, 17).

The Deep Darkness section of the Binkley Cave system is a temperate limestone karst cave in southern Indiana situated approximately 60 m below the surface, typifying permanent aphotic conditions, constant temperature, and minimal anthropogenic influence (18). These conditions produce severe attenuation of allochthonous organic inputs, making it one of the most energetically constrained microhabitats available for study in temperate North America. While temperate-zone caves in North America have received some attention (9–11, 19–21), few studies have simultaneously combined molecular, metabolic profiles, and environmental approaches across spatial gradients to examine deep, isolated cave zones, such as Deep Darkness. The Deep Darkness zone thus offers a natural experimental gradient for examining how carbon scarcity, sediment stoichiometry, and moisture collectively regulate microbial community composition and functional potential, with implications for cave conservation and karst hydrology. Detailed site geology and hydrogeology are provided in the Materials and Methods.

Prior to 1997, fewer than 40 publications addressed the microbial world of caves and karst environments (3, 16). The challenges associated with accessing sample sites, potential hazards, such as slippery surfaces and loose rocks, studying low-biomass samples, and reliance on cultivation-based techniques have contributed to the scarcity of data on cave microbial ecology. However, the advent of molecular phylogenetics in the late 1990s revealed the potential diversity of microbes in cave environments (6, 7, 19, 22). Our study integrates community-level physiological profiling (CLPP) and culture-independent sequencing to explore microbial adaptation in the Deep Darkness zone of Indiana Caverns (DDIC). We sought to, (i) characterize microbial community diversity in the DDIC, a system for which no molecular microbiological data currently exist, and identify enriched taxa relative to the surface environment and in other caves; (ii) assess functional diversity in carbon substrate utilization via CLPP assays, focusing on whether cave microbes preferentially metabolize complex/recalcitrant substrates (e.g., polymers, aromatics) over labile molecules (e.g., sugars, amino acids); and (iii) evaluate environmental drivers of functional variation, including sediment properties, and spatial position within the Deep Darkness. We hypothesize that (i) microbial communities will show phylogenetic novelty and dominance by oligotrophic or chemolithotrophic taxa adapted to low-carbon conditions, and (ii) these communities will selectively degrade complex substrates, reflecting long-term adaptation to low availabilities of labile carbon. We further predict that hydrologically connected zones will display elevated functional diversity or activity relative to more isolated cave zones, reflecting the influence of surface-derived organic inputs.

## MATERIALS AND METHODS

### Background and site description

**Fig 1:**
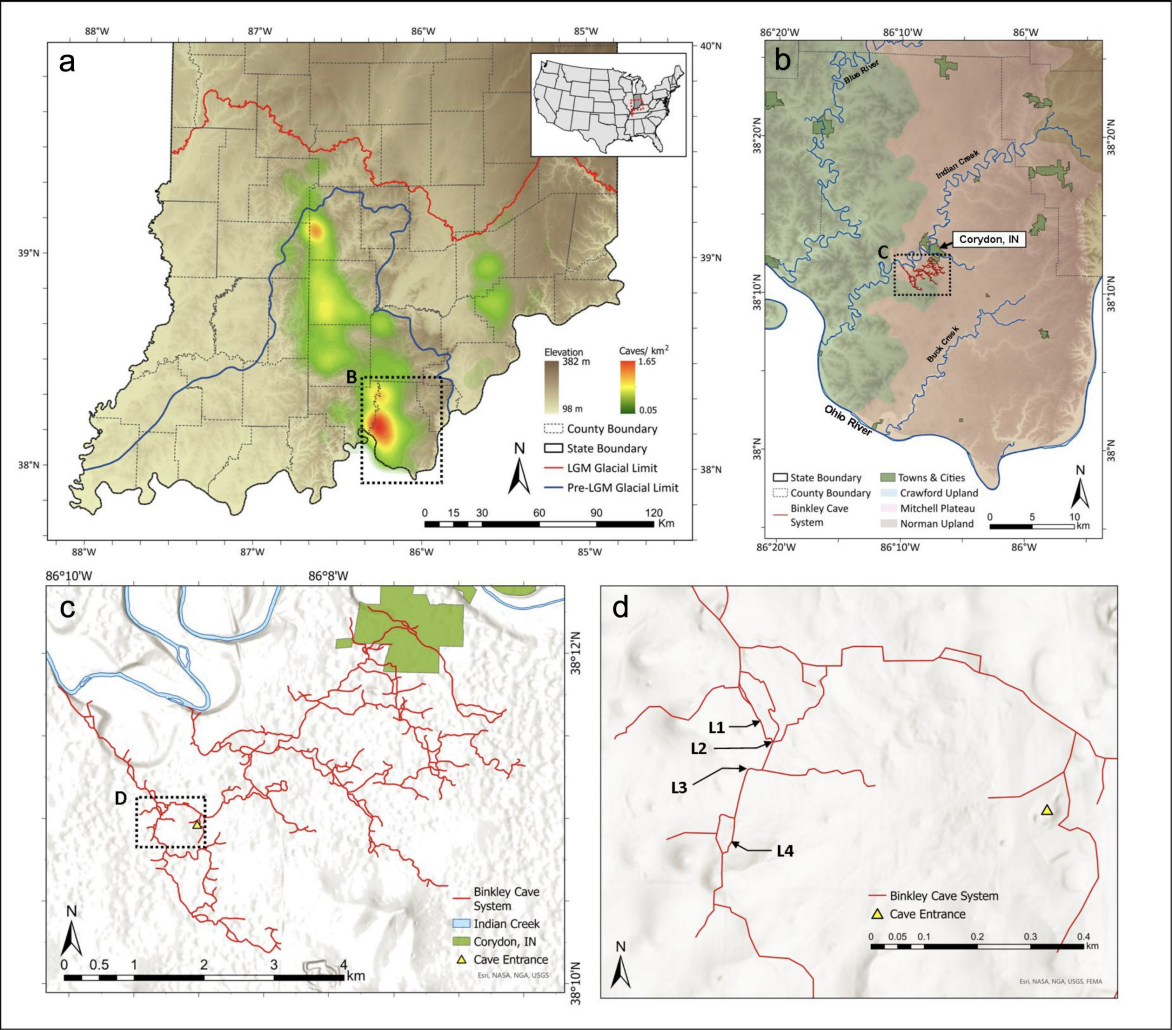
Maps of Indiana showing selected features. (**a**) Map showing elevation, the distribution of cave density (caves km⁻²) across southern Indiana (24, 25), and the last glacial maximum (LGM) and pre-last glacial maximum (Pre-LGM) limits. The small inset map indicates the continental United States with Indiana highlighted. The study area lies within the unglaciated karst region of south-central Indiana. (**b**) Map of Harrison County, Indiana, showing the location of the Binkley Cave system relative to Indiana’s physiographic provinces, surface topography, and major surface water systems (e.g., Indian Creek and the Ohio River). (**c**) Passage map of the Binkley Cave ystem indicating the Corydon entrance and major cave conduits. (**d**) Sampling locations within the cave showing sediment and water sampling sites L1 → L2 → L3 → L4 (proximity sequence from entrance to deepest point). Basemap layers in panels** a and b** were provided by the U.S. Geological Survey (USGS). Basemap layers in panels **c and d** were provided by Esri and include source data from Esri, NASA, NGA, USGS, and other contributing agencies.

The Binkley Cave system is located within the unglaciated physiographic provinces of southern Indiana (Fig. 1a and b). Specifically, the system is located within the Mitchell Plateau (23), which is host to numerous karst features (e.g., sinkholes, caves, springs, and sinking streams) that were formed by the dissolution of its Mississippian-aged (middle Mississippian) limestone bedrock. This province is bordered to the east by the lower Mississippian strata (siltstone and shale) of the Norman Upland, and to the west by upper Mississippian to lower Pennsylvanian strata (sandstone, shale, and limestone) of the Crawford Upland (Fig. 1b). The Binkley Cave system is formed primarily within the thinly bedded St. Louis limestone formation of the Blue River group, which is characterized primarily by limestone with locally significant beds of gypsum, anhydrite, shale, chert, and calcareous sandstones (14, 18).

Located south of Corydon, Indiana, the Binkley Cave system comprises two sections: the tourist section (Indiana Caverns, 38.18283°N, 86.15027°W) and the non-tourist section (Deep Darkness). This study focuses on the Blowing Hole segment of the DDIC. Situated around 60 m below the surface, this segment is characterized as a low anthropogenic section of the cave. Given its depth, the DDIC is subject to constant temperature (13.9°C water temperature), constant high humidity, and permanent aphotic conditions. The DDIC was discovered in 2009 by a group of cavers from the Indiana Speleological Society. The underground river in the Blowing Hole/Deep Darkness section of Binkley exits the cave via Harrison Spring on Blue River. We collected water and sediment samples aseptically from four distinct sites within the cave system: (i) location 1 (L1)—sediment (S1) and water (W1) samples were collected in an area characterized by a waterfall just beyond a 95-ft descent through a tunnel (access to the cave is through the blasted entrance that leads to the tunnel) and a subsequent 90-ft vertical drop into the cave; (ii) location 2 (L2)—sediment (S2) and water (W2) samples were collected from an isolated pool adjacent to collapsed roof rocks just before Rimstone Way; (iii) location 3 (L3)—sediment (S3) and water (W3) samples were collected from a deep pool located at a stream bend; and (iv) location 4 (L4)—sediment (S4) and water (W4) samples were collected from an isolated pool within the overflow route in the deepest accessible zone of the system (Fig. 1d). Thus, L1 is the most proximal and L4 the most distal site relative to the cave entrance. Reference sediment samples were collected (*n* = 3; approx 1–5 cm of topsoil in each) at the surface immediately outside the cave entrance, where forest grows directly above extensive cave and sinkhole systems. These samples were amalgamated and mechanically homogenized to form a single composite sample. All samples were kept on ice (4°C) during transport and processed within 24 h of collection; sub-samples for DNA extraction were stored at −80°C until analysis. Access to Indiana Caverns for research was granted by the cave management staff, and all activities were conducted on 21 September 2024, in the presence of an official cave representative and with explicit site permission.

### Environmental and biomass variables

A multiparameter, portable water probe (Hanna Instruments, USA) was used to measure water temperature (°C), conductivity (µS/cm), dissolved oxygen saturation (% DO), pH, and oxidation-reduction potential (mV ORP). At each location, water samples were collected in a sterile 500 mL plastic bottle, and four randomly distributed sediment subsamples (approx 1 m apart) were combined into one composite sample representing average site conditions. The top 5 mm of undisturbed sediment (approx 10 g) was carefully scraped and collected with minimal disturbance using stainless hand-held shovels. Samples were placed in sterile Whirl–Pak bags, stored on ice, and transported to the laboratory. In the laboratory, each composited soil sample was divided into subsamples to characterize soil total organic carbon (TOC), carbon, and nitrogen proportions (%C and %N) by direct combustion with a Thermo Flash 2000 Elemental Analyzer at the Fermentation Science Institute at Southern Illinois University. The microbial biomass (nmol PO_4_ gdw) quantified in triplicate using the phospholipid phosphate method (20) was determined spectrophotometrically (610 nm) using a SPECTROstar Nano microplate reader (BMG LABTECH, Ortenberg, Germany). Elemental analyses of soil and sediment samples were performed at DePauw University using a Bruker 5g X-Fluorescence spectrometer (p-XRF) (26). Elemental analysis was done in comparison to average Indiana soils of southern Indiana after data from Smith et al. (27). Percent soil water contents were determined in triplicate using BTS Precision Moisture Analyzer (Torbal, New York, USA).

### Community-level physiological profiling

Functional diversity within the samples was assayed using BIOLOG EcoPlate (28) as community-level physiological profiles. Prior to inoculation on the 96-well microplates, 2 g of sediment were suspended in a final volume of 30 mL of PBS solution, and inoculum densities were standardized at 0.3–0.5 optical density (OD) at 600 nm. A volume of 150 μL of the suspension was inoculated into each well in the EcoPlate, and all plates were placed in polyethylene bags to avoid desiccation and incubated aerobically. Water samples were directly inoculated onto the microplates. The 96-well EcoPlate containing 31 different carbon substrates (28) in triplicate was incubated at 25°C for 168 h, and color development was monitored every 24 h using a SPECTROstar Nano (BMG LABTECH) microplate reader at a wavelength of 590 nm. Parameters, such as average well-color development (AWCD), substrate richness (R), Shannon-Weaver diversity index (H), and Shannon substrate evenness (E), were calculated and used to analyze the carbon substrate utilization pattern. For each well (*i*), AWCD was calculated as ΣOD_i_/31 by averaging the absorbance of all substrate wells after subtracting the control, R was represented as the number of metabolized substrates, H = Σpi(lnpi), where pi = ODi/ΣOD_i_, and E was calculated as H/lnR. When OD_i_ *<* 0, the values were set to 0 to minimize bias (26, 28).

### DNA extraction and 16S rRNA gene amplicon sequencing

Total environmental DNA was extracted from frozen sediment samples using the Quick-DNA Fecal/Soil Microbe Midiprep Kit (Cat. No. D6110; Zymo Research, Irvine, CA, USA) following the manufacturer’s protocol. Approximately 3 g of each sample was processed, and DNA extracted from triplicate subsamples per location was pooled to obtain representative composite DNA samples. DNA concentration was quantified spectrophotometrically at 260 nm, and purity was assessed from absorbance ratios at 260/280 nm and 260/230 nm using an ND-2000 Nanodrop spectrophotometer (Thermo Fisher Scientific, Wilmington, DE, USA). Extracted DNA was stored at −20°C until sequencing. Normalized DNA (20 ng µL⁻¹) was submitted to GENEWIZ, Inc. (South Plainfield, NJ, USA) for 16S rRNA gene amplicon sequencing. The V3–V4 hypervariable regions of the 16S rRNA gene were amplified using a proprietary multiplexed primer set compatible with Illumina sequencing adapters. A second limited-cycle PCR was used to attach sample-specific dual indices and Illumina sequencing adapters for multiplexing. Indexed amplicons were pooled in equimolar amounts, purified, and subjected to quality control before sequencing. Libraries were sequenced on the Illumina MiSeq platform using 2 × 250 bp paired-end chemistry, ensuring complete coverage of the V3–V4 region. Raw reads were processed using QIIME 2 (29, https://docs.qiime2.org); forward and reverse reads were joined, demultiplexed by barcode, and trimmed of barcode and primer sequences. Sequences not meeting the following quality criteria were discarded: minimum length 200 bp, no ambiguous bases, and mean quality score ≥20. Chimeric sequences were identified and removed using the UCHIME algorithm (30) referenced against the RDP Gold database. Effective sequences were clustered into operational taxonomic units (OTUs) using VSEARCH v1.9.6 (31) against the Silva 119 database pre-clustered at 97% sequence identity. Taxonomic assignment of all OTUs was performed using the RDP Naïve Bayesian classifier (32) against the Silva 119 database (33) at a confidence threshold of 0.8. Sequences were rarefied prior to calculation of diversity statistics; alpha diversity was assessed using the Shannon index and Chao1 richness estimator, and beta diversity was calculated using weighted and unweighted UniFrac distances, with principal coordinate analysis (PCoA) and UPGMA clustering performed on the resulting distance matrices. Community composition was summarized across all taxonomic levels for each sampling location.

### Statistical analysis

One-way analysis of variance (ANOVA) or Kruskal-Wallis (for non-normal distribution), followed by Tukey’s highly significant difference or Dunn’s post hoc test for comparison of means, was performed for physico-chemical parameters, heavy metals, H-index, and total microbial biomass. Elemental averages of the sampled sites were calculated and normalized to the average of Indiana soils. The horizontal line in the plot area on a log scale (y-axis) is the average Indiana soil—values above this line are enriched in those elements, and values below this line are depleted in those elements relative to average Indiana soils.

To avoid selecting a specific time point or AWCD fixed level, a known limitation in CLPP analysis (34, 35), we analyzed the entire incubation data set following the approach of Perujo et al. (35). Absorbance values from all incubation times were treated as repeated measures after expressing all the data matrix as compositional data and expressed as isometric log-ratios. This method preserves the original data’s structure, enabling robust CLPP inference. We assessed differences by site and sample type using Type III repeated measures MANOVA with Wilks’ Lambda test statistic, modified from Perujo et al. (35). CLPP patterns were visualized in a canonical bivariate plot. Statistical significance was set at *P* < 0.05. All statistical analyses were performed in Python (version 3.9.6), Matplotlib (version 3.9.4), and Seaborn (version 0.13.2). Python libraries were used for data visualization.

Alpha-diversity metrics, including the Abundance-based Coverage Estimator (ACE), Chao1 richness, Shannon diversity index, and Simpson’s diversity index, were calculated using QIIME v1.9.1. ACE and Chao1 estimators were used to assess total bacterial richness, accounting for undetected taxa, whereas Shannon and Simpson indices quantified both richness and evenness within each community. To evaluate community dominance structure, core microbial dominance (the proportion of OTUs with relative abundance >1%) was computed to assess whether microbial assemblages at each cave location were dominated by a few highly abundant taxa or evenly distributed across multiple taxa. Relative abundances of taxa were calculated as percentages of total sequence counts per sample. Taxonomic distributions at the phylum and order levels were visualized using bar plots to illustrate compositional differences among sampling sites. Beta-diversity was evaluated using weighted UniFrac distances, which incorporate phylogenetic relatedness and relative abundance of OTUs to assess community similarity among samples. These distance matrices were used for hierarchical clustering (Ward’s method) and PCoA to visualize patterns of microbial community structure across cave and surface reference soil samples. To examine relationships between environmental variables and microbial community composition, principal component analysis (PCA) was performed on physicochemical parameters, allowing identification of environmental gradients most strongly associated with variation in community structure. All statistical analyses and data visualizations were performed in Python v3.9+ using the following libraries: pandas v1.5+, numpy v1.23+, scipy v1.9+, matplotlib v3.6+, seaborn v0.12+, scikit-learn v1.1+ (for various statistical data visualization, heatmap generation, data preprocessing, standardization, principal component analysis, etc.). Venn analysis diagram of shared and unique OTUs was constructed using a web-based tool, InteractiVenn, developed by Heberle et al. (36).

## RESULTS

### Physicochemical analysis of sediments and water from Deep Darkness Indiana Caverns

Physicochemical characteristics of water samples from DDIC are summarized in Table 1. Water temperature was consistent across sampling locations (13.97°C–14.02°C), with no significant spatial variation observed. The water was circumneutral in pH (7.37–7.45) and exhibited moderate electrical conductivity (458–572 μS/cm), consistent with values typical of natural surface waters. Dissolved oxygen (% DO) saturation was very low (3.8%–11%), indicating hypoxic conditions that may limit most aerobic aquatic life. This low % DO is also correlated with measured oxidation-reduction potential values ranging from 265.9 to 305 mV.

**TABLE 1:** Physicochemical properties of cave waters across sampling locations in the DDIC ecosystem

Location	mV ORP	pH	Dissolved oxygen (%)	Conductivity (µS/cm)	Temperature (°C)
WL 1	265.9	7.45	11.0	572	14.02
WL 2	291.2	7.37	3.8	524	13.98
WL 3	297.3	7.39	8.0	571	14.06
WL 4	305.0	7.40	7.4	458	13.97

**Fig 2:**
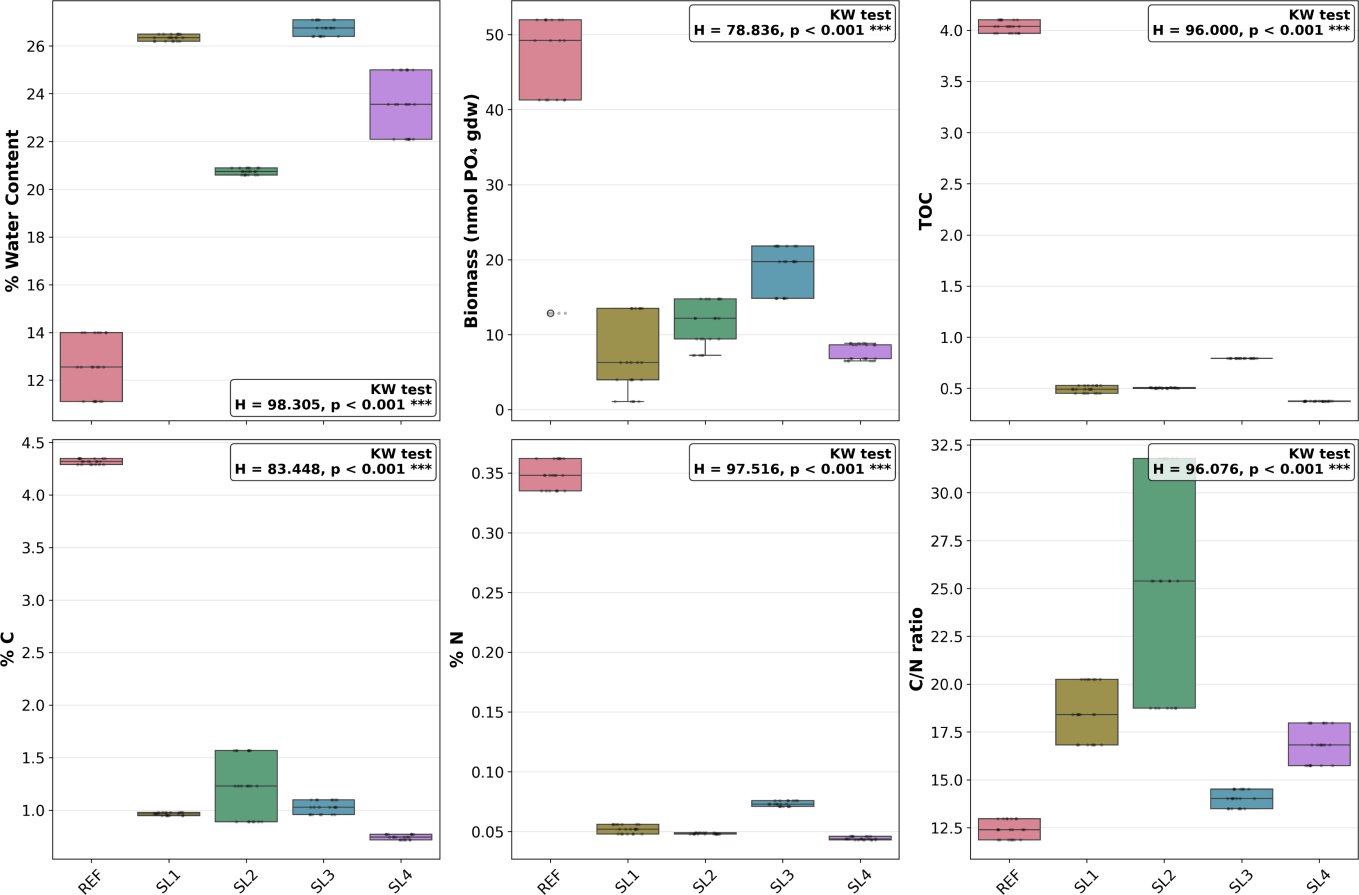
Boxplots representing measured soil properties at the reference surface soil (REF) and in the four cave locations (KW = Kruskal-Wallis test, *n* = 3). TOC = total organic carbon, %C = percent carbon, %N = percent nitrogen.

Multiple soil properties differed between the surface reference soil and cave sites, and in some cases among sampling sites within the cave. Percent water content was significantly higher in cave sediments compared to the surface reference soil (*P* < 0.001), with SL1 and SL3 showing the greatest water content among the cave sites (Fig. 2). Microbial biomass was significantly greater in the surface reference soil (40.94 ± 14.62 nmol PO₄ gdw⁻¹) than in the cave (6.56 ± 4.86 to 18.80 ± 2.99 nmol PO₄ gdw⁻¹). Similarly, TOC, percent carbon, and nitrogen concentrations were highest in the surface reference soils and lowest within the cave (Fig. 2). In contrast, the C:N ratio was significantly lower at the surface reference soil compared to cave sites, except for SL3. Notably, C:N ratios also differed significantly among cave sites (e.g., SL2 vs SL3 and SL4).

**Fig 3:**
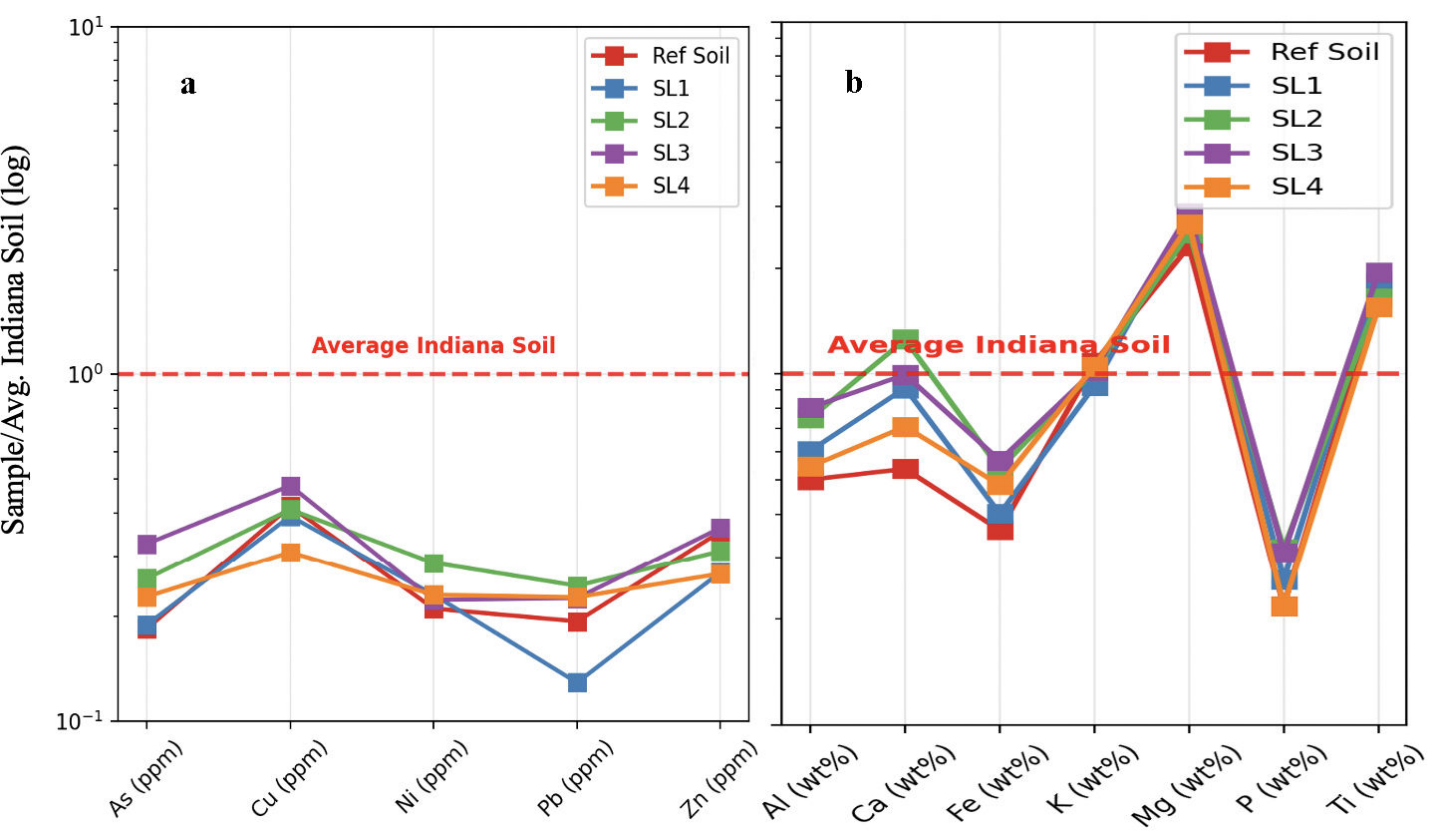
Mean normalized elemental concentrations, (**a**) in trace elements (ppm), and (**b**) in major elements (wt%) found in sampled cave sediments and reference soil compared to the Indiana state average (red dashed line) for regional soils (27).

Elemental concentrations in sediments determined by X-ray fluorescence spectroscopy are shown in Fig. 3. Major (e.g., Al, Ca, Fe, K, Mg, P, and Ti) and trace element (As, Cu, Ni, Pb, and Zn) concentrations were normalized to average topsoil compositions from southern Indiana (27). Cave sediment samples consistently show higher concentrations of Mg and Ti (wt%), lower concentrations of Al, Fe, and P (wt%), and comparable concentrations of K (wt%) to the average southern Indiana topsoil composition. Calcium (wt%) shows considerable variation in the cave sediments, which does not correlate with sample location. Cave sediments contain trace element concentrations that are consistently below concentrations in average topsoil compositions. Correlation analysis showed positive associations among major elements and trace elements, including Al–P (r = 0.87, *P* = 0.055), Al–Ti (r = 0.85, *P* = 0.068), Al–Fe (r = 0.91, *P* = 0.032), Ti–Ni (r = 0.96, *P* = 0.010), and Ti–Zn (r = 0.93, *P* = 0.022), whereas Pb exhibited weak-to-moderate negative, non-significant correlations with most elements (e.g., Pb–Ti: r = −0.74, *P* = 0.156; Pb–Mg: r = −0.61, *P* = 0.270), indicating geochemical behavior distinct from the other metals.

### Carbon utilization in surface reference soil and cave bacterial communities

**Fig 4:**
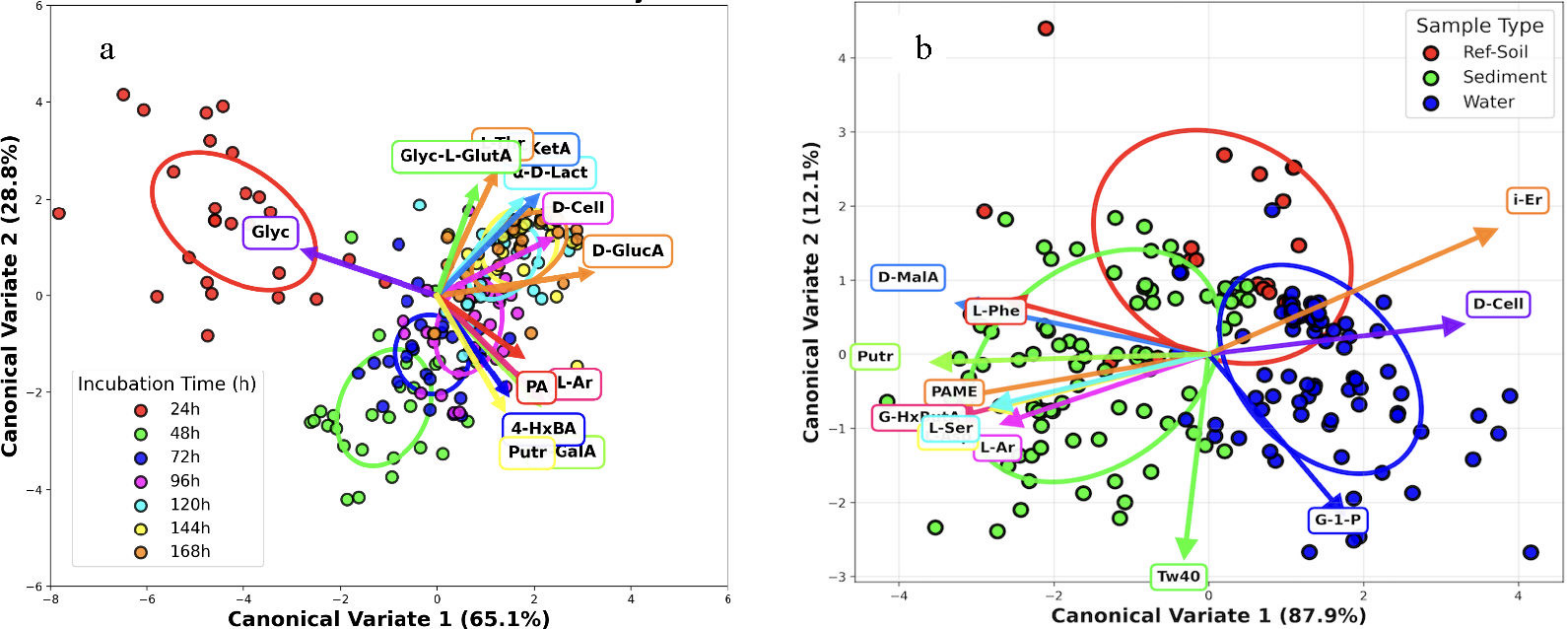
Canonical variates plot based on cave CLPP data expressed as compositional data, (**a**) temporal changes across the incubation period. Metabolic profiles at 24 h were distinct due to glycogen utilization, while later time points (48–168 h) showed a transition from amines/amides and carboxylic acids toward carbohydrates and amino acids, (**b**) differences by sample type. Can1 (≈87.9%) separated water column CLPPs from sediments, with the reference site intermediate along Can1 and separated by Can2 (≈12.1%). Ellipses grouped most of the samples.

Figure 4a illustrates temporal changes in cave CLPPs across the incubation period. Canonical variate 1 (x-axis) accounted for most of the variation (65.1%), with an additional 28.8% explained by the y-axis. At 24 h, metabolic fingerprints were clearly distinct from later incubation points, primarily driven by the degradation of glycogen (Glyc), a complex carbohydrate derivative. A temporal trend was evident along the x-axis from 48 to 168 h, reflecting a shift in substrate utilization. Early incubation was characterized by preferential metabolism of phenylethylamine and putrescine (amines/amides), D-galacturonic acid and 4-hydroxybenzoic acid (carboxylic and acetic acids), and L-arginine (amino acid). By contrast, later incubation was associated with utilization of L-threonine, β-hydroxy-glycyl-L-glutamic acid (amino acid), α-D-lactose and D-cellobiose (carbohydrates), α-ketobutyric acid, and D-glucosaminic acid (carboxylic and acetic acids).

Type III repeated-measures MANOVA revealed significant differences in CLPPs across incubation times, indicating that metabolic profiles shifted over the course of the experiment. Significant differences were also observed among sample types (Table 2, *P* < 0.001; Fig. 4b). However, the interaction between incubation time and sample type was not significant (Table 2, *P* = 0.167). The canonical biplot based on sample type (Fig. 4b) revealed that bacterial communities clustered primarily according to the habitat (sediment vs water), Can1 explained most of the separation (≈87.9%), distinguishing cave water column CLPPs (positive component) from sediment CLPPs (negative component), while the reference site clustered near the center along Can1 but was separated along Can2 (≈12.1%). Substrate preferences also differed by habitat: bacterial communities in the cave water disproportionately utilized carbohydrates, such as i-erythritol, D-cellobiose, and glucose-1-phosphate, whereas cave sediment communities exhibited broader substrate use spanning carbohydrates (e.g., pyruvic acid methyl ester), carboxylic and acetic acids (D-malic acid, γ-hydroxybutyric acid), amines/amides (putrescine), and amino acids (L-phenylalanine, L-serine, L-arginine).

**TABLE 2:** Type III repeated measures MANOVA test of ilr-coordinates of CLPP data from incubation of cave water and sediment samples and reference forest soil at the cave entrance^*a*^

Effect	Wilks' Lambda	F	df1	df2	*P* value	Sig.^*b*^
Sample type	0.1335	8.1523	62	291	0.0000	***
Incubation time	0.0648	2.7544	186	869	0.0000	***
Sample type × incubation time	0.0904	1.0790	372	1,680	0.1677	

^*a*^ The analysis utilized ilr (isometric log-ratio) coordinates to properly handle the compositional nature of the substrate data.

^*b*^ ***P < 0.001.

**Fig 5:**
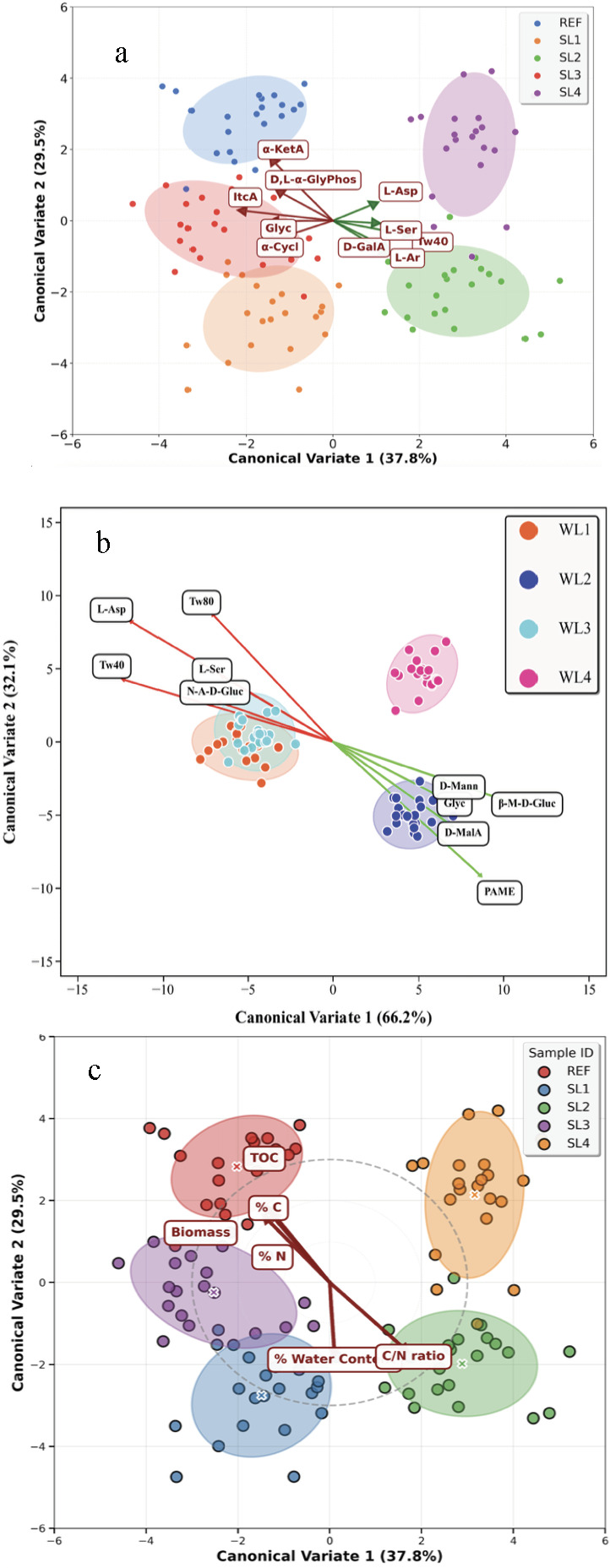
Canonical analysis of CLPPs from (**a**) surface reference soils, cave sediments, and (**b**) cave water samples, and the 10 most influential carbon substrates across samples, (**c**) the effects of biomass and soil parameters on the sedimentary CLPP of cave bacterial community and reference surface soil.

As statistical analyses showed significant differences in CLPPs among sample types in MANOVA analysis, CLPPs were plotted by sample types. Figure 5a shows spatial heterogeneity and distinct CLPPs clusters within the cave and at the reference site, indicating divergent substrate utilization patterns and significant spatial variation in microbial community functional profiles across cave locations. Surface communities preferentially metabolize α-ketobutyric acid (carboxylic and acetic acids) and D,L-α-glycerol phosphate (carbohydrates). In contrast, cave sediment CLPPs varied by site: for example, site SL2 was characterized by higher oxidation of L-arginine and L-serine (amino acids), D-galacturonic acid (carboxylic and acetic acids), and Tween 40 (polymer), whereas site SL3 exhibited preferential metabolism of itaconic acid (carboxylic and acetic acids), α-cyclodextrin, and glycogen (polymers). Figure 5b depicts clustering of WL1 and WL3 water samples, which overlapped in functional profiles, while WL2 and WL4 separated spatially. WL1 and WL3 were distinguished by preferential oxidation of Tween 40 and Tween 80 (polymers), L-asparagine and L-serine (amino acids), and N-acetyl-D-glucosamine (carbohydrate). By contrast, WL2 communities showed higher utilization of pyruvic acid methyl ester, D-mannitol, and β-methyl-D-glucoside (carbohydrates), D-malic acid (carboxylic and acetic acids), and glycogen (polymer). Canonical variate analysis of soil biogeochemical properties revealed significant spatial heterogeneity across the sampled locations (Fig. 5c). The first two canonical variates explained 67.3% of the total variance (CV1: 37.8%; CV2: 29.5%), demonstrating clear discrimination among sample groups. Reference samples were strongly associated with elevated TOC, %C, %N, and microbial biomass. SL1 samples were distinctly positioned in the lower-left quadrant and primarily correlated with increased water content, indicating moisture-driven differentiation. SL2 samples formed a separate cluster in the lower-right quadrant with a strong positive correlation to the C/N ratio, reflecting distinct organic matter quality and decomposition state. SL4 samples occupied the upper-right quadrant with minimal association to measured soil factors. The clear spatial separation and distinct vector associations demonstrate that soil biogeochemical properties vary significantly across the study area, with organic matter content, moisture levels, and nutrient stoichiometry serving as primary drivers of microbial habitat differentiation.

### Community composition and diversity of surface reference soil and cave microbiomes

**Fig 6:**
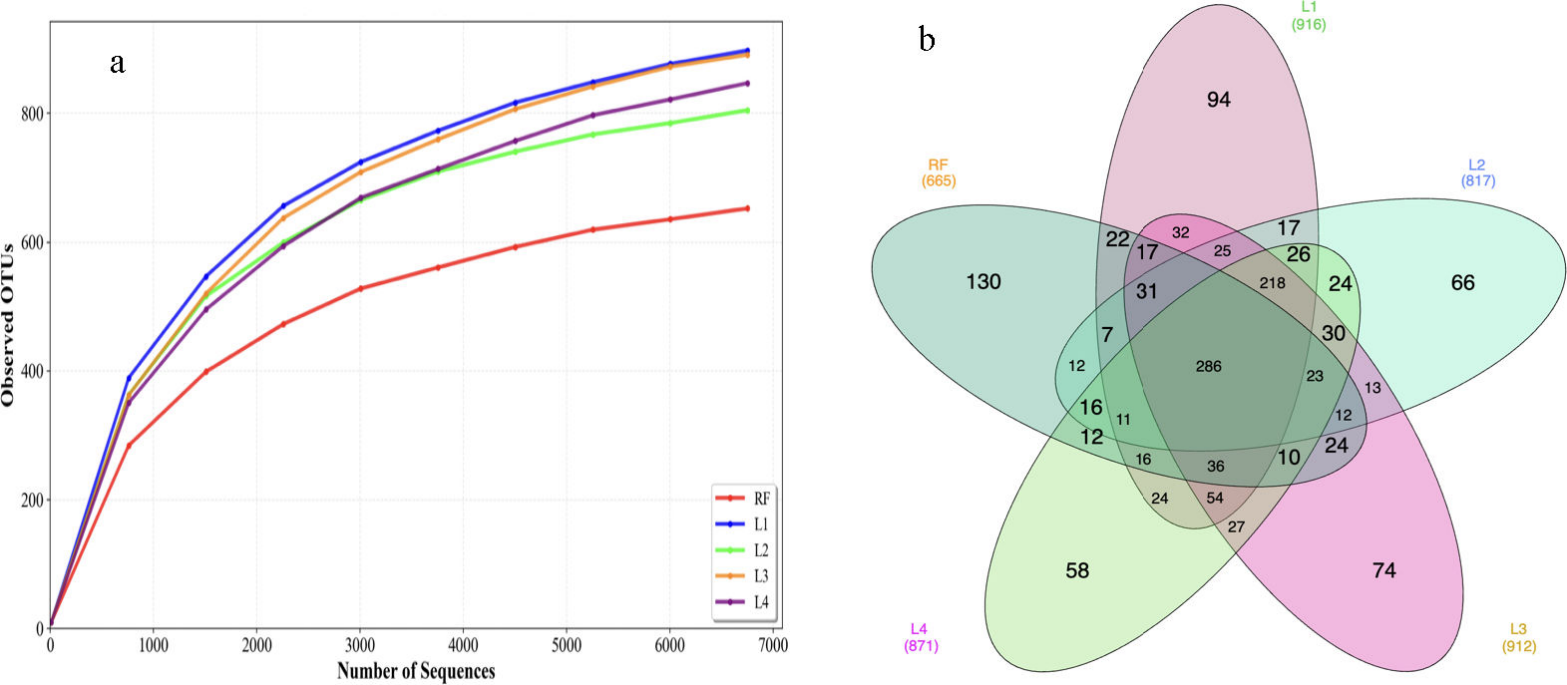
(**a**) Rarefaction curves indicating the number of detected OTUs as more sequences are considered per sample, and (**b**) Venn diagram of the exclusive and shared OTUs of the surface reference soil (RF) and four locations in the cave.

A total of 51,103 paired-end reads per sample (range: 45,503–55,901) (Table 3) were obtained after merging forward and reverse reads. Following quality filtering and chimera removal, 35,832–42,874 high-quality non-chimeric sequences were retained per sample (68%–79% effective rate). These non-chimeric reads were used for subsequent OTU clustering and taxonomic analyses. The sample from surface reference soil had the highest number of aligned reads (42,874), while L4 had the lowest number (35,832). A rarefied analysis of the sequenced products (Fig. 6a) showed that the cave community demonstrates a significantly higher species richness and diversity than that found in the surface reference soil sample, despite a significant reduction in the amount of available organic carbon and biomass (Fig. 2). Indeed, the ACE–Chao1 comparison (Table 3) indicates that microbial richness increases from surface reference soil to deeper cave locations (L3–L4). The close agreement of ACE and Chao1 at RF and L1 suggests sufficient sampling (data not shown), while higher Chao1 estimates at deeper cave sites suggest greater unseen diversity and a higher prevalence of rare taxa, consistent with environmental filtering and niche specialization in subterranean ecosystems. Shannon and Simpson indices were consistently high across all sites (≥8.5 and ≥0.99, respectively) (Table 3), indicating that even community structures were dominated by no single taxon. Good’s coverage values (≥0.97) confirm that sequencing depth was sufficient to capture most of the community diversity at each site (Table 3).

**TABLE 3:** Comparative alpha diversity metrics of microbial communities from cave habitats and reference soil

Sample	Reads	ACE	Chao1	Shannon	Simpson	Good coverage
RF	55,901	743.846	761.037	7.982	0.992	0.983
L1	52,637	1,011.633	1,006.311	8.816	0.995	0.978
L2	45,617	882.079	886.574	8.692	0.995	0.983
L3	51,777	1,022.005	1,011.21	8.72	0.995	0.976
L4	45,503	1,020.952	1,036.924	8.552	0.994	0.972

In terms of substrate richness (functional diversity), surface soil samples exhibited the highest values (mean ± SD: 21.71 ± 9.60), reflecting access to a diverse array of carbon substrates. Sediment samples showed moderate richness (range: 16.43–19.33), while water samples displayed considerable variability: WL1 (22.38 ± 10.16) and WL3 (22.05 ± 10.10) were comparable to surface soil levels, whereas WL4 exhibited markedly reduced richness (1.43 ± 1.12) (KW: H = 65.08, *P* < 0.0001). Shannon diversity followed a similar pattern, with RF, WL1, and WL3 displaying high functional diversity indicative of broad and even substrate utilization. In contrast, WL4 showed very low Shannon diversity, reflecting a highly restricted metabolic profile (KW: H = 61.41, *P* < 0.0001) (data not shown).

Across the RF and cave sites (L1–L4), we detected 1,472 OTUs in total. OTU richness ranged from 665 OTUs in RF to 916 OTUs in L1, with the remaining cave sites containing 817 (L2), 912 (L3), and 871 (L4) OTUs (Fig. 6b). A core microbiome of 286 OTUs was shared among all sites, representing taxa capable of persisting across the surface-to-subterranean environmental gradient. Pairwise comparisons indicated substantially greater OTU sharing among cave sites than between the cave and surface soil. RF shared 426–439 OTUs with cave locations, whereas cave locations shared 621–699 OTUs with one another. Correspondingly, Jaccard similarity coefficients were considerably lower between RF and cave sites (0.36–0.39) compared to within-cave comparisons (0.56–0.62). The strongest similarity occurred between L3 and L4 (J = 0.622), both situated deeper within the aphotic zone. Despite the presence of a shared core, each site also contained unique OTUs. RF harbored 130 OTUs not detected within the cave system. In contrast, L1–L4 contained fewer unique OTUs (94, 66, 74, and 58, respectively).

**Fig 7:**
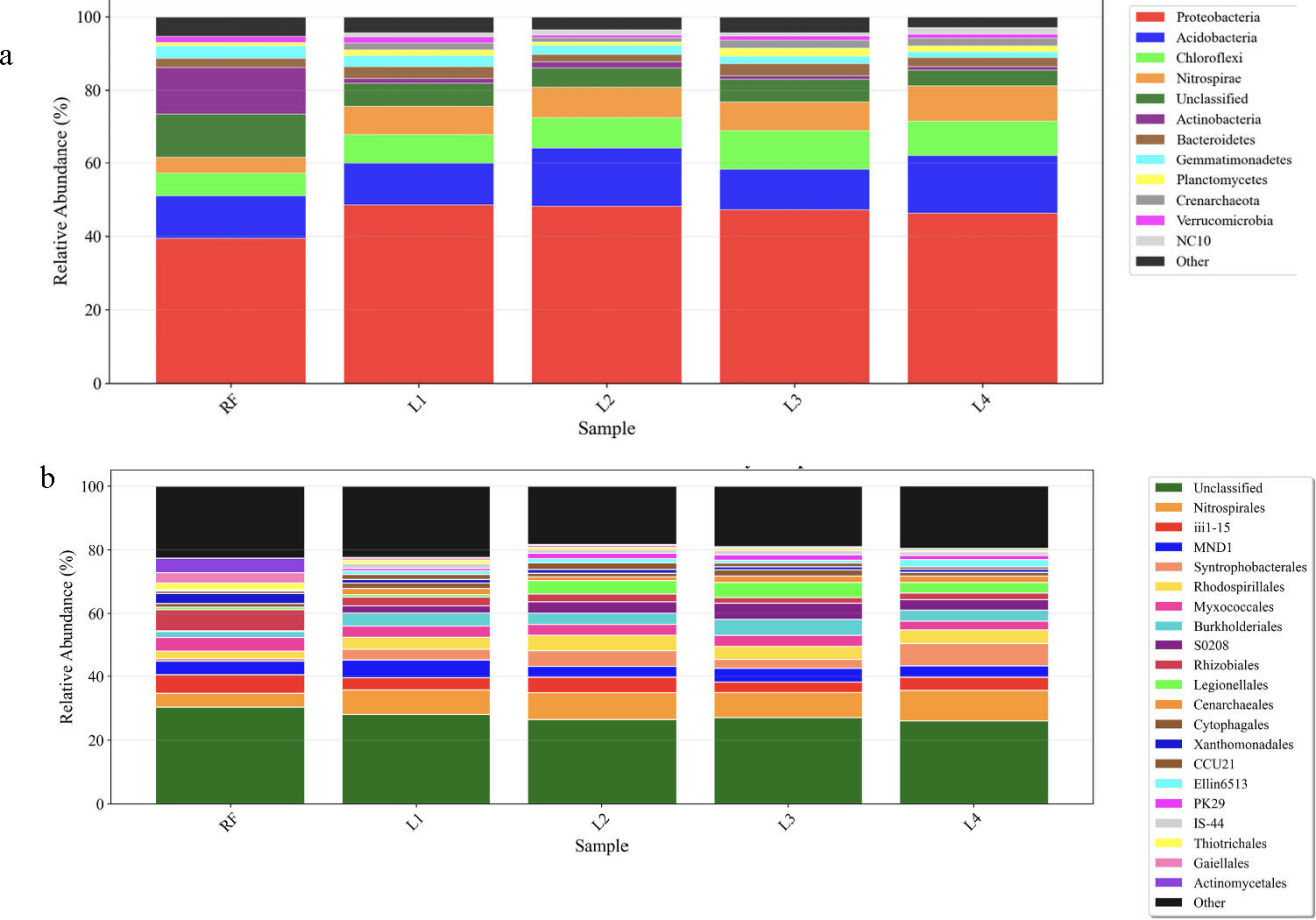
Relative abundance of dominant (minimum 1% of the community) (**a**) bacterial phyla and (**b**) order level for cave and surface reference soil (RF) samples. All other phyla less than 1% were merged into the “Other” group. The relative abundance of each bacterial phylum refers to the proportion of reads that aligned to OTUs associated with the phylum/order in each sample.

Figure 7a shows the relative abundances of the top 11 bacterial phyla in all samples. Both surface soil and cave zone bacterial communities were dominated by several main bacterial phyla (e.g., Proteobacteria, Acidobacteria, and Chloroflexi); however, the relative abundances of the majority of the bacterial phyla were higher in the cave sites compared to the surface reference soil: Proteobacteria (mean relative abundance RF: 39.49% vs Cave: 47.62%), followed by Acidobacteria (RF: 11.64% vs Cave: 13.59%), Chloroflexi (RF: 6.11% vs Cave: 9.13%), Nitrospirae (RF: 4.35% vs Cave: 8.38%), Bacteroidetes (RF: 2.41% vs Cave: 2.75%), Planctomycetes (RF: 0.97% vs Cave: 1.62%), NC10 (RF: 0.03% vs Cave: 1.25%), and an archaeal phylum: Crenarchaeota (RF: 0.0% vs Cave: 1.83%). In contrast, the relative abundance of bacterial phyla, such as Actinobacteria (RF: 12.74% vs Cave: 1.2%), Gemmatimonadetes (RF: 3.52% vs Cave: 2.27%), Verrucomicrobia (RF: 1.55% vs cave: 1.16%), and unclassified bacterial sequences (RF: 11.96% vs Cave: 5.49%) were higher in the surface soil compared to the cave sites (Fig. 7a).

**Fig 8:**
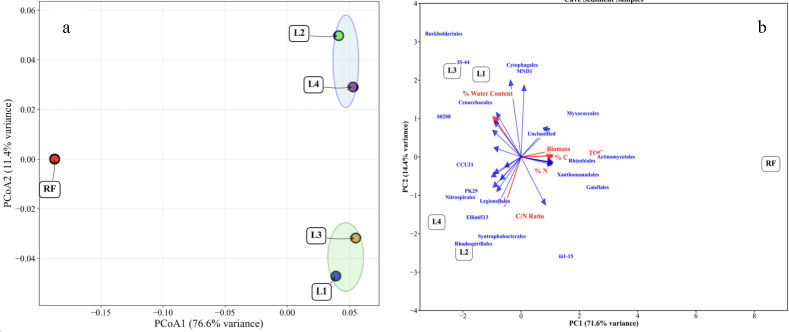
(**a**) PCoA plot of weighted Unifrac distance matrix of the sediment samples of Indiana Caverns Deep Darkness and reference soil sample (RF). First, two PCs explained 88% of the variation (shown in parentheses). RF had the most divergent microbial composition, whereas L1 and L3, and L2 and L4 had a more similar community composition. (**b**) PCA biplot showing correlation between physicochemical factors of sediment samples with the dominant bacterial orders. Red vectors represent physicochemical factors (% water content, %C, %N, C/N ratio, biomass, TOC) and blue vectors represent the top bacterial orders. Sample locations are shown in boxes.

The relative abundance of bacterial orders varied slightly between the RF and the cave locations, although the overall community structure remained broadly consistent across samples (Fig. 7b). In the RF sample, unclassified taxa accounted for approximately 30% of the total community, representing the most dominant fraction (Fig. 7), followed by Rhizobiales (6.64%), Nitrospirales (4.35%), Actinomycetales (4.6%), Xanthomonadales (3.15%), Rhodospirillales (2.35%), Burkholderiales (1.84%), and Syntrophobacterales (0.79%). These orders collectively contributed to a highly diverse microbial assemblage, consistent with the heterogeneity and nutrient variability characteristic of surface soils. In contrast, the cave samples (L1–L4) exhibited a slight decrease in the proportion of unclassified taxa (26.9%), accompanied by increases in Nitrospirales (8.38%), Syntrophobacterales (4.51%), Rhodospirillales (4.44%), Burkholderiales (4.02%), and Legionellales (3.23%), suggesting a gradual shift toward microbial taxa adapted to the more stable and oligotrophic cave conditions. Cytophagales (1.15%–1.95%) and Myxococcales (2.79%–4.55%) remained relatively low but consistently abundant across all sampling sites. PCoA based on Bray-Curtis dissimilarity revealed distinct clustering patterns between the RF and the cave samples (L1–L4) (Fig. 8a). The first two axes explained 76.6% and 11.4% of the total variance, respectively, capturing the major gradients in community composition. The microbial community from the RF clustered separately from all cave-associated samples along the first coordinate axis, indicating substantial compositional differentiation between the surface and subsurface environments. In contrast, the cave samples (L1–L4) formed two closely related clusters: one comprising L1 and L3, and another containing L2 and L4. The proximity of samples within each cluster suggests high similarity in community composition among cave locations, with minor spatial variation likely reflecting microenvironmental differences such as moisture, substrate type, or localized nutrient inputs. PCA biplot further revealed strong correlations between bacterial community composition and measured environmental variables across cave and reference soil samples (Fig. 8b). The first two axes explained 71.6% and 14.4% of the total variance, respectively, indicating that most of the community variation could be attributed to gradients in carbon and nutrient availability. The RF separated distinctly along the first axis, corresponding to higher TOC, %C, and microbial biomass, which were positively associated with Actinomycetales, Rhizobiales, and Xanthomonadales. In contrast, cave locations (L1–L4) clustered toward the opposite side of the ordination, reflecting reduced nutrient conditions and distinct microbial associations. Sites L1 and L3 grouped closely and were positively correlated with higher water content and the presence of Burkholderiales, Cytophagales, and *MND1*. Conversely, L2 and L4 were positioned toward lower carbon and nitrogen gradients, aligning with taxa such as Rhodospirillales, Syntrophobacterales, and Nitrospirales (Fig. 8b).

## DISCUSSION

Subterranean ecosystems are among the most nutrient-limited habitats on Earth (16, 37, 38), yet the ecological strategies that sustain microbial life under persistent energy scarcity remain poorly understood. To examine how these constraints shape microbial community assembly and function, we performed an integrated 16S rRNA gene-based metagenomic survey and carbon substrate utilization assay across spatial and physicochemical gradients in the DDIC ecosystem. Sediment properties shifted markedly from the surface into the cave, with sharp declines in biomass, TOC, and nitrogen, accompanied by increases in water content and elevated C:N ratios, reflecting progressive organic matter depletion and altered nutrient stoichiometry with distance from the entrance. Across this gradient, we detected 42 bacterial phyla, with Proteobacteria, Acidobacteria, Chloroflexi, and Nitrospirae consistently enriched alongside a notable fraction of unclassified lineages consistent with previous studies on bacterial communities within caves (11, 19, 22). Despite strong resource limitation, cave communities remained phylogenetically diverse yet exhibited clear spatial organization and shifts in substrate utilization breadth, suggesting that microbial persistence in Deep Darkness is governed not simply by low carbon availability, but by interactions between hydrologic connectivity, sediment chemistry, and metabolic specialization. Together, these findings reveal that even in extreme environments where energy inputs are minimal (Fig. 2) (7), microbial communities maintain unexpected phylogenetic breadth while reorganizing around resource-efficient metabolic guilds that enable long-term persistence.

### Environmental controls on microbial community assembly in Deep Darkness

Across the Deep Darkness cave transect, sediments and water data collectively indicate a strongly oligotrophic, low-energy environment. Cave sediments exhibited pronounced reductions in microbial biomass and edaphic factors relative to surface soil, with a concurrent increase in water content and C:N ratio deeper into the cave (Fig. 2). These shifts reflect diminished allochthonous organic inputs and slow nutrient turnover, characteristic features of carbon-limited subterranean ecosystems (7, 16, 37). Water chemistry was consistently circumneutral and oxidizing, with moderate dissolved oxygen, indicating that aerobic and microaerophilic metabolisms are supported, but energetic supply is constrained. Conductivity values (458–572 μS/cm) fall within the range associated with autogenic recharge and short epikarst flow paths (15, 39), suggesting low ionic enrichment and minimal influx of nutrient-bearing groundwater, reinforcing the interpretation of limited hydrologic connectivity. Importantly, trace and major element concentrations in cave sediments fell within or below southern Indiana State soil baselines (Fig. 3), showing no evidence of metal toxicity or geochemical enrichment that might otherwise shift community structure via stress selection. The absence of elevated inhibitory trace metals (e.g., Pb, Ni, Cu) indicates that energy limitation, rather than metal stress, is the dominant ecological constraint in this system (40). The elevated Ca and Mg concentrations are consistent with carbonate bedrock dominated by calcite and dolomite. Dissolution of these minerals is the primary source of Ca²^+^ and Mg²^+^ in cave waters and sediments and strongly influences cave geochemistry by buffering pH and regulating alkalinity, with important implications for microbial colonization and biogeochemical cycling (1, 16, 41). Additional enrichment may arise from the weathering of minor accessory minerals embedded within the carbonate matrix, which contribute residual cations during progressive dissolution (39, 42). Phosphorus likely derives from a combination of organic inputs, particularly guano and surface-derived detritus, as well as the weathering of phosphate-bearing minerals such as apatite (43, 44); however, the low P concentrations observed in the cave sediments relative to average topsoil compositions suggest limited contributions from both sources. The elevated Ti concentrations may be attributed to clay minerals weathered out of the thinly bedded limestone layers or from minor Fe–Ti oxide minerals (e.g., magnetite or ilmenite) weathered from local beds of shale and calcareous sandstone commonly found in the St. Louis Limestone (14, 18). It is also possible that the elevated Ti concentrations reflect some input from insoluble detrital material, including Ti-bearing clays or residual mineral phases introduced via soil infiltration or surface inputs (39). Regardless of the source, the correlation between Al and Ti (r = 0.85) suggests that the Ti is likely stored within the clay minerals found in the cave sediments. Although Ti is biologically inert, it may serve as an indicator of clay-rich sediment that indirectly structures microbial habitats by increasing surface area and nutrient sorption capacity (45, 46). Thus, microbial community assembly is shaped primarily by nutrient scarcity and carbon flow, rather than by toxicity-driven filtering in this cave.

Microbial biomass and TOC were significantly greater in the surface reference soil than in the cave. This pattern is consistent with higher inputs and assimilation of organic matter in surface habitats that support greater plant and animal biomass (8, 47). These environmental gradients corresponded to systematic restructuring of microbial communities. Carbon-rich surface soils supported heterotrophic decomposers, including *Actinomycetales*, *Xanthomonadales*, *Rhizobiales*, and *Acidobacteriales*, taxa associated with carbohydrate and carboxylic acid degradation (e.g., α-ketobutyric acid, and D,L-α-glycerol phosphate) and rhizosphere C-cycling (48–51). In contrast, deep cave sites characterized by the lowest microbial biomass and TOC, and highest C:N ratios, were dominated by *Planctomycetota*, *Caulobacterales*, *Nitrospirales*, *Burkholderiales*, *Bdellovibrionales*, and *Sphingomonadales*, reflecting biofilm-based carbon retention, chemolithotrophic energy acquisition, slow growth, and microbial predation (3, 43, 44). Biomass, %C, %N, TOC, and C:N ratio were the strongest predictors of community structure (Fig. 5c and 8b), while water chemistry explained additional variation, underscoring that resource scarcity, not geochemical stress, drives microbial biogeography in this cave.

### Functional adaptation and carbon-source utilization

Surface reference soil communities exhibited greater metabolic versatility and preferentially oxidized labile substrates, including a wide range of carbohydrates, carboxylic acids, and amino acids, classified as low molecular weight organic substances (LMWOS: e.g., D,L-α-glycerol phosphate, α-ketobutyric acid, i-erythritol in the Ecoplate) (Fig. 5a); patterns that align with richer organic inputs, plant-derived litter, and higher microbial turnover in surface environments (52, 53). In contrast, cave communities clustered separately and showed increased reliance on complex or recalcitrant carbon sources, such as polymers in addition to oxidizing labile substrates (Fig. 5a through c). Ecoplate substrates, such as Tween-40, Tween-80 (in water communities), α-cyclodextrin, and glycogen (in sediment communities), were more heavily utilized at oligotrophic cave sites (e.g., SL1, SL3) (Fig. 5a and b), consistent with metabolic strategies typical of subsurface microbes that persist under severe carbon limitation (16, 38). The heterogeneity among cave sites, such as SL2 and SL4, displaying modest uptake of select labile compounds (e.g., amino acids) compared to moisture-rich locations (SL1 and SL3), and the preferential oxidation of α-cyclodextrin, itaconic acid, and glycogen, suggests that microhabitat variation in water content and organic matter deposition influences functional metabolic potential (54). Collectively, these CLPP results indicate that surface soils support copiotrophic and metabolically flexible communities, whereas cave sediments harbor diverse microbial assemblages specialized for LMWOS, slow growth, and the decomposition of energy-poor substrates.

### The Deep Darkness microbiome and environmental filtering

Analysis of 16S rRNA amplicon sequences revealed that microbial communities in the DDIC encompass a broad phylogenetic diversity, including representatives from multiple established bacterial phyla, as well as a fraction of unclassified taxa typical of subterranean ecosystems. When comparing bacterial phylum-level composition between the cave and surface soils, and relative to other cave systems, Proteobacteria consistently dominate cave sediment communities across geographically and geologically distinct systems: 48.24% in a low-anthropic-activity cave in northwestern Romania (6), 32.9% ± 7.5% in nutrient-limited subarctic caves (7), and approximately 20% in lava caves at Lava Beds National Monument (55), with our DDIC value of 47.6% falling within this range and most closely resembling the Romanian cave system. Acidobacteria contributions are similarly consistent across studies; 8.18% (6), 17.8% ± 4.0% (7), and ~10% (20), compared to 13.6% in DDIC, confirming Acidobacteria as a broadly distributed oligotrophic cave taxon. Chloroflexi 9.1% contribution in DDIC is closely similar to 7.9% ± 3.5% in subarctic caves (7). Nitrospirae were detected at 1.9% ± 1.0% in subarctic caves (7) and ~7% in lava caves (55), consistent with our observation of 8.3% in DDIC sediments, suggesting a conserved chemolithotrophic niche for this phylum across cave types.

Our results diverge most strikingly from published cave studies in the near absence of Actinobacteria. Actinobacteria are typically among the dominant phyla in oligotrophic cave ecosystems, comprising ~39% in lava cave microbial mats (55), ~28%–60% on speleothem surfaces in Kartchner Caverns (20), 10.0% ± 3.8% in subarctic caves (7), and 12.6% in Romanian karst caves (6). In sharp contrast, Actinobacteria represent only 1.2% of the DDIC microbiome, among the lowest values reported for any temperate cave system. This unusually low abundance likely reflects the near-complete absence of refractory organic substrates, such as lignin and humic compounds, that typically sustain Actinobacterial decomposers in cave environments with stronger surface connectivity (21). Instead, DDIC deeper sediments are enriched in *Gallionellales*, *Crenotrichales*, *Pirellulales*, and *Gemmatales*; taxa commonly associated with chemolithoautotrophy, mixotrophy, and carbon-conserving metabolic strategies, suggesting that the DDIC operates under a distinct energy regime where chemolithotrophic and oligotrophic metabolic guilds replace the decomposer-dominated communities characteristic of better-connected cave systems. Interestingly, the archaeal phylum Crenarchaeota appeared at ~2% abundance in cave sediments yet was absent in the surface reference soil, consistent with observations of elevated cave-specific archaeal signals in karst and deep cave zones where Thaumarchaeota and Crenarchaeota dominate under nutrient-poor, aphotic conditions (38, 56–58), suggesting that Crenarchaeota may represent a specialized subsurface lineage adapted to the extreme oligotrophy of the Deep Darkness zone. This pattern suggests that the Deep Darkness environment preferentially supports ammonia-oxidizing Archaea adapted to extreme oligotrophy (57, 58). The co-occurrence of Crenarchaeota with Nitrospirales further indicates a coupled archaeal-bacterial nitrification process that may constitute a key autotrophic energy pathway in this system. Compared to other cave studies that report <2% archaeal abundance (59, 60), the relative prominence of Crenarchaeota in DDIC suggests that nitrogen chemolithotrophy may play a disproportionately important role in sustaining microbial productivity where organic inputs are severely limited. Future work with archaeal‐specific primers, metagenomics, and transcriptomics could validate whether these Crenarchaeota populations contribute significantly to nitrogen cycling or alternative energy pathways in this nutrient-limited cave ecosystem.

Community composition clearly differentiated surface soils from cave sediments, reflecting strong environmental filtering along gradients of carbon availability and moisture. The surface reference soil clustered with high TOC and elevated microbial biomass and was enriched in heterotrophic bacterial orders, such as *Actinomycetales*, *Rhizobiales*, *Myxococcales*, *Phycisphaerales*, *Pirellulales*, *Rhodospirillales*, and *Acidobacteriales*. These taxa include aerobic decomposers, rhizosphere-associated groups, and metabolically versatile heterotrophs that thrive in organic-rich environments and efficiently utilize a wide range of carbohydrates and plant-derived inputs (61–65). In contrast, cave sites (SL1/SL3; SL2/SL4) clustered along PCA axes (Fig. 8b) associated with higher water content and elevated C:N ratios; environmental signatures of deeper, oligotrophic cave sediments.

The SL1/SL3 cluster was enriched in oligotrophic and chemolithotrophic taxa, including *Cytophagales*, *Cenarchaeales*, and *Sphingomonadales* (*Novosphingobium* group), alongside the predatory lineage *Anaeromyxobacteraceae*-affiliated *Myxococcales* (64), while the SL2/SL4 cluster was enriched in a functionally diverse assemblage encompassing chemolithotrophs (*Nitrospirales*), biofilm-associated oligotrophs (*Pirellulales*, *Planctomycetales* OR-59-associated, *Burkholderiales*, *Legionellales*, *Sphingomonadales*), and the obligate predatory lineage *Bdellovibrionales* (66), collectively reflecting the co-occurrence of multiple trophic strategies across both clusters. These taxa are frequently reported from nutrient-poor subterranean systems where energy conservation, mineral-associated metabolisms, and reduced-carbon substrate utilization are favored (38, 67, 68). Many of these oligotrophic groups contain members capable of microaerophilic iron oxidation, dissimilatory metal reduction, and polymer or complex organic matter degradation (2, 68–72), traits well aligned with the low-organic, mineral-rich conditions characteristic of karst cave sediments.

The relatively low but consistent abundances of *Cytophagales* and *Myxococcales* across all sampling sites are consistent with prior cave studies, which likewise report these groups as persistent but minor constituents of subterranean communities (7, 73, 74). *Cytophagales* are known degraders of recalcitrant polymers, such as cellulose and chitin (75), whereas *Myxococcales* function as bacterial predators that recycle microbial biomass under nutrient limitation (7, 76, 77). Their recurrence across microhabitats suggests that polymer degradation and microbial predation remain stable ecological processes in the Deep Darkness, even under severe carbon limitation. Such patterns further indicate strong environmental filtering that maintains small but functionally important guilds within the broader cave metacommunity.

Overall, the taxonomic shifts observed between the surface soil and cave clusters indicate that environmental filters, particularly carbon limitation, organic matter inputs, and sediment moisture, strongly structure microbial communities within the cave, giving rise to functionally and phylogenetically distinct assemblages across microhabitats (16, 54). These findings mirror broader patterns observed across karst systems globally, where resource heterogeneity and microhabitat structure strongly influence microbial functional potential and phylogenetic composition (7, 67).

Surprisingly, across all sequencing-based diversity metrics, microbial communities inhabiting the Deep Darkness zone exhibited greater taxonomic richness than the organic-rich surface soil. Rarefaction curves and richness estimators (ACE, Chao1) showed that each cave site contained more observed and predicted OTUs than the surface soil, while Shannon diversity was likewise higher in all subterranean samples. This pattern contrasts with the traditional expectation that oligotrophic cave ecosystems harbor reduced diversity and instead supports emerging evidence that subterranean microbial communities can be highly diverse when niche partitioning, microhabitat heterogeneity, and slow-growth dynamics reduce competitive exclusion (16, 67). Similar findings have been reported in other karst environments, where gradients in moisture, mineral surface soil chemistry, and redox potential create a mosaic of ecological niches that support many low-abundance specialists (7, 20, 54).

Despite the regional connectivity typically expected between surface soils and underlying karst environments, we detected a relatively small, shared core microbiome of 286 OTUs (19.8% of total richness across all sites). This level of overlap is comparable to the 11%–16% shared OTUs commonly reported from other subterranean habitats, where strong environmental filtering and low-energy conditions limit the persistence of many surface-derived taxa (20, 55). Although some cave systems show greater similarity to exterior soils, for example, a 21% OTU overlap between speleothem or damp ceiling communities and surface soils (78), our observed overlap remains substantially lower than the 58.3% similarity that Thompson et al. (78) reported for cave sediment communities directly influenced by surface inputs. Likewise, Porca et al. (79) found highly similar microbial profiles across three geographically distant European limestone caves, suggesting that some cave systems host broadly conserved microbiota. In contrast, the comparatively low shared OTU fraction in the DDIC indicates that although dispersal from the surface likely contributes to community assembly, strong species sorting associated with oligotrophy, elevated moisture, and mineral-surface microhabitats acts as a dominant ecological filter. Consequently, only a subset of surface-derived taxa can establish or persist in this environment (e.g., *Cytophagales* and *Myxococcales*), resulting in a microbiome that is taxonomically distinct and shaped primarily by subterranean environmental constraints rather than mass effects alone.

### Contrasting hydrologically connected and isolated cave zones

Our prediction that hydrologically connected zones would exhibit elevated functional diversity or activity relative to more isolated cave zones received partial support. In the water column, hydrologically active sites (WL1 and WL3), located beneath a waterfall recharge zone and along an active stream bend, displayed higher OTU richness and broader carbon-substrate utilization profiles than the more isolated WL2 and WL4 sites. This pattern is consistent with studies showing that surface-derived organic inputs delivered through dripping water, waterfalls, or stream flow can enhance microbial activity and functional diversity in cave pools (12, 38, 80). However, this trend was not uniformly observed in sediment communities, where functional and taxonomic diversity were more strongly associated with local physicochemical properties, particularly TOC, microbial biomass, and C:N ratios, rather than hydrological connection. In line with our observations, previous studies report that microbial communities in karst caves and sinkholes are primarily structured by environmental filters, particularly gradients in nutrients, organic carbon, moisture, and mineralogy, rather than by hydrological connectivity alone. These physicochemical constraints are well documented as key drivers of microbial assembly in oligotrophic subterranean ecosystems (16, 54, 81, 82). Thus, while hydrological connectivity appears to enhance functional diversity in aquatic microhabitats, sediment communities are predominantly shaped by resource limitation and geochemical constraints, resulting in partial support of the hypothesis.

The four sampling locations capture the principal hydrological and physicochemical gradient of the Blowing Hole segment, from active recharge zones near the entrance to isolated oligotrophic pools in the deepest accessible zone, but cannot fully resolve spatial heterogeneity across several km of passage. Although the Deep Darkness lacks speleothem surfaces and guano deposits (simplifying its microhabitat landscape relative to many cave systems), fine-scale heterogeneity driven by moisture regime, sediment texture, and proximity to hydrological flow paths persists, as evidenced by the L1/L3 and L2/L4 community clustering in the PCoA (Fig. 8a). Within-site variance also cannot be formally estimated given the composited sampling design. Future studies should expand site coverage across contrasting hydrological conditions, incorporate seasonal replication, full water geochemical analysis (IC, ICP-MS), and process within-site replicates independently. Such expanded sampling would also support machine learning approaches to quantitatively model environmental-microbiome relationships, an analytical direction that remains underpowered in the present data set.

### Conclusion

This study characterized microbial diversity and function in sediments and water of an oligotrophic temperate cave with minimal anthropic impact. Microbial communities in the Deep Darkness zone exhibited notably high phylogenetic diversity and clear spatial structuring consistent with environmental filtering along physicochemical gradients. Carbon availability, nutrient stoichiometry, and sediment moisture emerged as dominant filters shaping both community composition and functional potential. Surface reference soil supported copiotrophic, metabolically versatile heterotrophs, whereas cave sediments and waters were enriched in oligotrophic and chemolithotrophic taxa adapted to carbon scarcity, with CLPP analyses revealing a corresponding shift toward complex and recalcitrant substrate utilization. The small core microbiome (19.8% shared OTUs) underscores strong species sorting, with only a subset of surface-derived taxa persisting under subterranean conditions. Cave communities diverged from typical cave systems in their exceptionally low Actinobacteria abundance and enrichment of chemolithotrophic and carbon-conserving lineages (e.g., *Gallionellales*, *Crenotrichales*, *Pirellulales*, *Gemmatales*), indicating an unusually severe energy-limited regime. Hydrological connectivity enhanced functional diversity in aquatic microhabitats, while sediment communities were governed primarily by local physicochemical constraints. Collectively, these findings refine ecological models of subterranean ecosystems by demonstrating that microbial persistence arises through interactions among microhabitat heterogeneity, metabolic specialization, and differential energy pathways rather than carbon limitation alone. Future work combining metagenomics, metatranscriptomics, and stable isotope probing would elucidate active metabolic pathways, while longitudinal sampling across hydrological seasons would clarify links between recharge dynamics and functional diversity.

## Data Availability

Extended data tables and supplementary analyses are available from the authors upon request, as they are not included in the published article. Raw 16S rRNA gene amplicon sequencing data have been deposited in the NCBI Sequence Read Archive (SRA) under BioProject: PRJNA1463864 (accession numbers: SRR38485895, SRR38485896, SRR38485897, SRR38485898, SRR38485899).
